# Robustness of STDP-induced memory to perturbations of presynaptic activity: a simulation study

**DOI:** 10.1186/1471-2202-12-S1-P290

**Published:** 2011-07-18

**Authors:** Youwei Zheng, Lars Schwabe

**Affiliations:** 1 Adaptive and Regenerative Software Systems, Department of Electrical Engineering and Computer Science, University of Rostock, Rostock, 18051, Germany

## 

Memory is believed to be stored in the patterns of synaptic connections, which have to be plastic in order to allow for learning new memories, but also stable to retain previously learned patterns (plasticity vs. stability). Previous work has shown that the so-called “additive” STDP rule leads to memory retention times, which are orders of magnitudes longer than achievable with a “multiplicative” rule [1]. Here we investigate a related consequence of STDP in model neurons for memory retention, namely the extent to which transient perturbations in presynaptic activity (mimicking paroxysmal activity) may affect previously learned memories.

We simulate a conductance-based one-compartment spiking model neuron with (“additive” and “multiplicative”) STDP learning rules for the excitatory (but not the inhibitory) synapses. Presynaptic activity is modeled via Poisson spike trains. We first let the distribution of synaptic weights converge to a steady-state. Then, the effects of transient changes in excitatory presynaptic activity are characterized in terms of the cross-correlation of the subsequent time-dependent synaptic weight patterns relative to a reference time just before the perturbation.

We find that even after moderate and short transients (10 to 15 Hz, < 1 min), a neuron may completely remain silent due to synaptic learning within the transient period, where strong synapses become weak. If a neuron does not remain silent, both magnitude and duration of the perturbation determine the scale of memory destruction, which was generally more severe for the multiplicative rule. As abnormal activity in epilepsy may be due to the changes of GABAergic functions, we also studied the effect of an increased GABAergic reversal potential reported in epilepsy. Surprisingly, increased reversal potentials (less inhibition) can benefit the robustness of memory as studied here.

Our results demonstrate that in model neurons without other homeostatic mechanisms, paroxysmal activity could easily override memories. This suggests that pathological brain states may be even more disruptive than previously thought when learning mechanisms are also taken into account.
